# Translational investigation and treatment of neuropathic pain

**DOI:** 10.1186/1744-8069-8-15

**Published:** 2012-03-09

**Authors:** Bo Xu, Giannina Descalzi, Hai-Rong Ye, Min Zhuo, Ying-Wei Wang

**Affiliations:** 1 Department of Anesthesiology, Xinhua Hospital, Shanghai Jiaotong University School of Medicine, Shanghai 200092, China; 2 Department of Physiology, Faculty of Medicine, University of Toronto Centre for the Study of Pain, Medical Sciences Building, 1 King's College Circle, Toronto, ON M5S1A8, Canada

## Abstract

Neuropathic pain develops from a lesion or disease affecting the somatosensory system. Translational investigations of neuropathic pain by using different animal models reveal that peripheral sensitization, spinal and cortical plasticity may play critical roles in neuropathic pain. Furthermore, descending facilitatory or excitatory modulation may also act to enhance chronic pain. Current clinical therapy for neuropathic pain includes the use of pharmacological and nonpharmacological (psychological, physical, and surgical treatment) methods. However, there is substantial need to better medicine for treating neuropathic pain. Future translational researchers and clinicians will greatly facilitate the development of novel drugs for treating chronic pain including neuropathic pain.

## Introduction

Neuropathic pain develops from a lesion or disease affecting the somatosensory system [1]. Triggers for neuropathic pain are numerous and diverse. It can be caused by direct and indirect injury to nerve systems. The classification of neuropathic pain is often based on the anatomical location of neurologic involvement (central or peripheral). Major forms of clinical neuropathic pain are given in Table 1. It is estimated that neuropathic pain afflicts millions of people worldwide [2]. Neuropathic pain reduces the patients' overall health-related quality of life (sleep, mood, work, social and recreational capacities), and generates health-care costs several times higher than in control groups [3]. However, the management of patients with chronic neuropathic pain is of utmost difficulty, and the response to existing treatments is often inadequate. Therapy for neuropathic pain includes the use of both pharmacological and non-pharmacological (psychological, physical, and surgical treatment) methods. The objectives of this review are to outline the underlying mechanisms and current therapies, and to discuss the recent advances that should be useful to guide the treatment of neuropathic pain in the future.

**Table 1:** Major forms of clinical neuropathic pain

Classification	Example
**Peripheral NP**	Postoperative neuralgia
	Post-traumatic neuralgia
	Incarcerated neuropathy (carpal tunnel syndrome)
	Phantom limb pain
	Painful polyneuropathy
	Trigeminal neuralgia
	Painful diabetic neuropathy
	Nutritional deficiency-related neuropathy
	Chemotherapy-induced neuropathy
	Radicular neuropathy (cervical, thoracic or lumbar
	vertebrae)
	Postherpetic neuralgia
	HIV neuropathy
	Pain associated with Guillain-Barre' syndrome
	Idiopathic sensory neuropathy
	Complex regional pain syndrome
	Tumors caused by nerve compression and leakage
**Central NP**	Post-stroke pain
	Spinal cord injury pain
	Spinal ischemia pain
	Syringomyelia
	Pain associated with multiple sclerosis
	Parkinson's disease-related pain

## Neuropathic pain

According to the new definition by IASP, neuropathic pain is a type of chronic pain caused by a lesion or disease of the somatosensory nervous system. Lesion means the directly damage to somatosensory system, while disease refers to indirectly injury by metabolic stress, autoimmune conditions or inflammatory and so on [1]. Such damage or lesion can take place not only somatosensory nerves, but also those innervating visceral organs. Hence, neuropathic pain is an aberrant somatosensory processing that contrasts with the normal plasticity of somatosensory system in nociceptive pain. According to the location of injury sites, neuropathic pain can be further divided into central neuropathic pain and peripheral neuropathic pain. Central neuropathic pain is pain caused by a lesion or disease of the central somatosensory nervous system; while peripheral neuropathic pain is caused by a lesion or disease of the peripheral somatosensory nervous system. Despite the different lesion or damage sites, central sensory synapses are likely involved in both cases.

In order to distinguish from neuropathic pain, nociceptive pain is also proposed by IASP to cover pain that arises from actual or threatened damage to non-neural tissue and is due to the activation of nociceptors. This new terminology, however, is less used in present literature.

## Basic mechanisms of neuropathic pain

Translational research involving animal models of neuropathic pain aims to identify molecular targets for the treatment of patients with chronic neuropathic pain. Major animal models are given in Table 2. Many recent studies employ chronic constriction injuries [4,5], spared nerve ligation [6,7] or common peroneal nerve ligation (CPN) [8,9], which include injury of, but not severing of peripheral nerves. Nerve ligation models have served as powerful tools that not only mimic clinical symptoms of chronic neuropathic pain, but also result in robust molecular and cellular alterations. For example, in humans chronic neuropathic pain is manifested through spontaneous pain that can be induced through innocuous stimuli (allodynia) [10]. Accordingly, mice and rats exposed to nerve ligation surgery display reduced mechanical thresholds, whereby previously innocuous mechanical stimuli elicit nociceptive paw withdrawal responses after surgery [11,12]. Similarly, chronic neuropathic pain patients also display exaggerated responses to noxious stimuli (hyperalgesia), and animal studies have shown similar responses following peripheral nerve injury [13]. Several tools have been devised to investigate these behavioral effects, most notably Von Frey filaments for mechanical allodynia, and the Hargreves test for thermal hyperalgesia. In this manner, animal studies have begun to identify molecular pathways that undergo robust changes in correspondence with the development of chronic neuropathic pain.

**Table 2:** Major animal models for the study of neuropathic pain

Classification	Name of model
**Peripheral NP**	Neuroma model
	Chronic constriction injury model (CCI)
	Partial sciatic nerve ligation model (PSL or Seltzer
	model)
	L5/L6 spinal nerve ligation model (SNL)
	Spared nerve injury
	Sciatic cryoneurolysis model (SCN)
	Inferior caudal trunk resection model (ICTR)
	Sciatic inflammatory neuritis model (SIN)
	Postherpetic neuralgia model (PHN)
	Diabetic neuropathic pain model
	Drugs-induced peripheral neuropathy model
	Bone cancer pain models
	HIV-induced neuropathy model
	Trigeminal Neuralgia model
	Orofacial pain model
**Central NP**	Excitotoxic spinal cord injury (ESCI)
	Photochemical SCI model
	Weight-drop or contusive SCI (Allen's Model)
	Spinal hemisection

**Figure 1:**
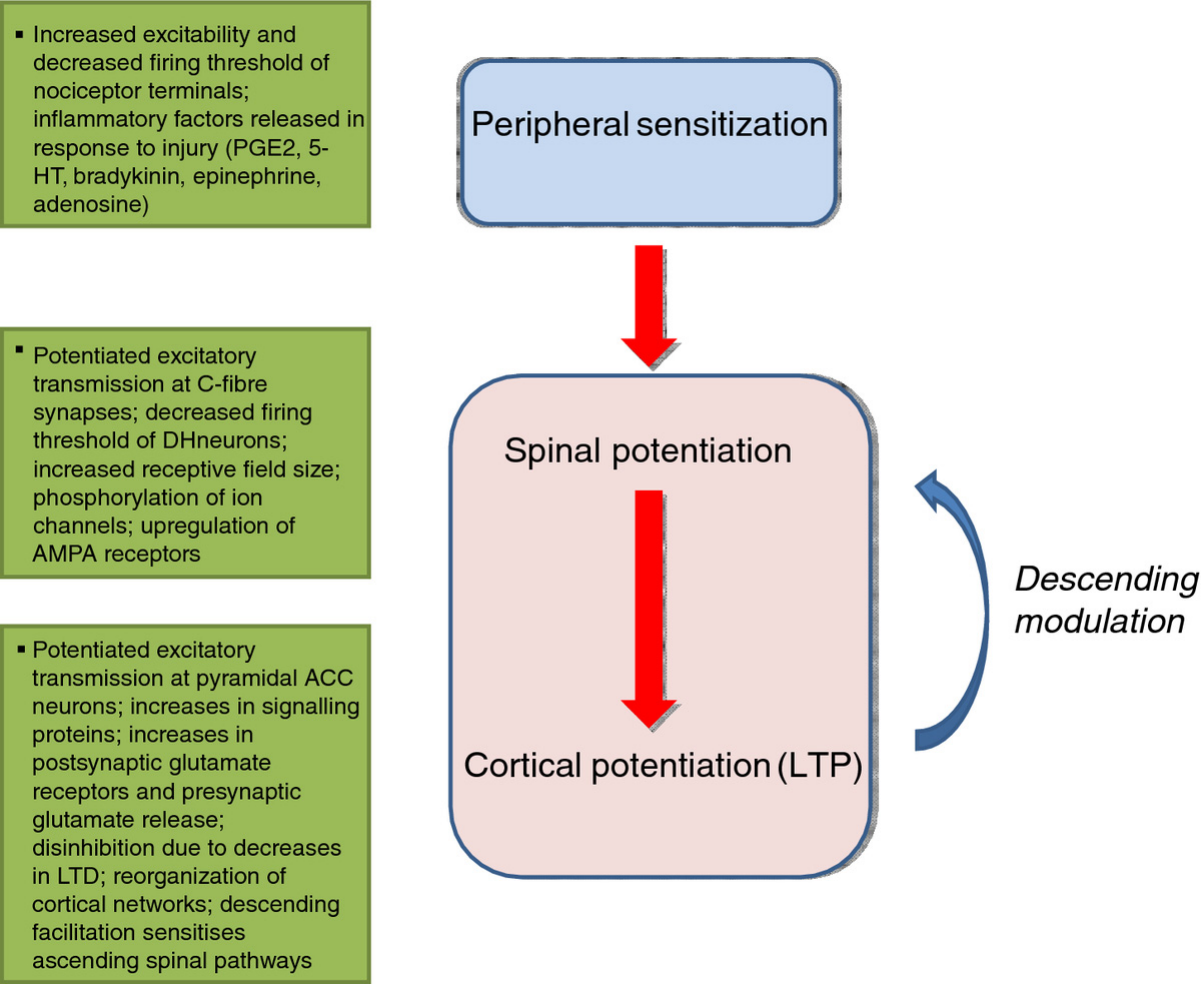
**Neuropathic pain is manifested through alterations at the peripheral, spinal, and cortical levels**. At the periphery, neuropathic pain is associated with changes in the excitability of nociceptors. At the spinal cord level, long-term potentiation (LTP) of sensory excitatory synaptic transmission take place at least at similar time scales. The upregulation of postsynaptic AMPA receptors including possible recruitment of silent synapses contribute to spinal LTP. Similar to LTP reported in other central synapses, different protein kinases and possible new protein synthesis are also required. Within the cortex, neuropathic pain is associated with the induction of LTP including late-phase LTP (L-LTP) at cortical synapses. Both presynaptic and postsynaptic alterations have been observed that result in ongoing potentiated excitatory activity. Dis-inhibition of local inhibitory modulation have been also found within the spinal dorsal horn and cortical areas. Descending faciltiatory modulation from cortical and sub-cortical areas are also thought to contribute to enhanced sensory transmission in the spinal cord dorsal horn.

Through the use of animal models, various molecular and cellular alterations have been identified in correspondence with chronic neuropathic pain. At the periphery, neuropathic injuries trigger sensitization and can induce long-term abnormal neural activity along primary afferent pathways [14] (see Figure 1). In the spinal cord, dorsal horn neurons display potentiated excitatory responses and decreases in firing threshold in response to chronic neuropathic pain [15-17]. For example, repetitive squeezing of the sciatic nerve induces long term potentiation (LTP) at synapses of C-fibre [17], and spinal activity has been found to be potentiated in chronic pain settings, whereby previously sub-threshold synaptic input is able to drive action potentials in dorsal horn neurons [10]. Nevertheless, pharmacological interventions targeting spinal level alterations fail to adequately terminate chronic pain, are often accompanied by adverse side effects, and decrease in efficacy over time. Consequently, recent investigations have focused on molecular changes within brain regions that may mediate chronic neuropathic pain. Interestingly, synaptic plasticity within key cortical areas involved in pain has been observed in correspondence with chronic neuropathic pain [12]. Specifically, studies consistently provide robust evidence of changes in excitatory transmission in animal models of neuropathic pain. Within the anterior cingulate cortex (ACC) for example, a critical brain region involved in pain affect [18], intracellular signaling cascades activated by peripheral neuropathic injury have been found to induce persistent molecular changes that potentiate glutamatergic excitatory transmission [9,19,20]. For example, it was shown to induce phosphorylation of AMPA GluR1 channels and enhance GluR1 mediated postsynaptic responses within the mouse ACC after nerve injury [9]. Interestingly, calcium-stimulated adenylyl cyclase 1 (AC1) was critical for this effect. Accordingly, AC1 mediated protein kinase M-ζ (PKMζ) activity was found to maintain neuropathic pain induced alterations within the ACC [21]. Specifically, *in vitro *ACC slice recordings revealed that ζ-pseudosubstrate inhibitory peptide (ZIP) inhibition of PKMζ prevented synaptic potentiation induced by nerve injury, whilst *in vivo *intra-ACC injections of ZIP significantly reduced mechanical allodynia. These observations allowed for the recent finding that specific inhibition of AC1 reduces chronic pain in animal neuropathic pain models [22]. Additional brain regions also have been observed to undergo changes in excitatory transmission, including the amygdala [23], insular cortex, and primary and secondary sensory cortices [14].

## Pharmacological management for neuropathic pain

The most effective clinical management to treat neuropathic pain is pharmacological therapy. However, only 40-60% of the patients achieve clinically meaningful pain relief with pharmacotherapy, since some kinds of neuropathic pain may be insensitive to ordinary analgesics. To improve the current treatment of patients with neuropathic pain, evidence-based studies should be made for the pharmacological therapies that are used clinically. Recently, numerous clinical researches have confirmed the efficacy of antiepileptics, antidepressants, opioid analgesics, and topical lidocaine [24] in some cases of neuropathic pain. In addition, several novel drug treatments such as botulinum toxin, capsaicin patch and lacosamide have been also used for neuropathic pain therapy. Table 3 summarizes the major pharmacological treatments for neuropathic pain and their analgesia mechanisms.

**Table 3:** Major pharmacological treatment for neuropathic pain and their basic mechanisms

Compound	Mode of action
**Antidepressants**	
Nortriptyline	Inhibition of both serotonin and norepinephrine reuptake
Desipramine	Inhibition of both serotonin and norepinephrine reuptake
Duloxetine	Inhibition of both serotonin and norepinephrine reuptake
Venlafaxine	Inhibition of both serotonin and norepinephrine reuptake
**Anticonvulsants**	
Gabapentin	Decreases release of glutamate, norepinephrine, and substance P, with ligands on α2-δ subunit of voltage
Pregabalin	Decreases release of glutamate, norepinephrine, and substance P, with ligands on α2-δ subunit of voltage
Lacosamide	Decreases release of presynaptic transmitters, inhibition of voltage-gated sodium-channel.
**Opioid agonists**	
Morphine	μ-receptor agonism
Oxycodone	μ-receptor agonism
Methadone	μ-receptor agonism,κ-receptor antagonism
Levorphanol	μ-receptor agonism
Tramadol	μ-receptor agonism, inhibition of norepinephrine and serotonin reuptake
**Topical therapy**	
5% lidocaine patch	Block of sodium channels
High-dose capsaicin patch	Damage of nociceptive sensory axons, a highly selective activating ligand for TRPV1,.
Botulinum toxin.	Inhibition of both the exocytosis of acetylcholine and some other neurotransmitters

### Antiepileptics

Gabapentin and pregabalin are antiepileptics that have been recommended as first-line drugs for neuropathic pain treatment. They selectively bind to the α2δ1 subunit of calcium channels in various regions of the brain and the superficial spinal dorsal horn. As a result, it inhibits the release of glutamate, norepinephrine, and substance P [25]. Both drugs have been found to provide effective pain relief in postherpetic neuralgia (PHN), painful diabetic peripheral neuropathy (PDN) as well as central pain [24]. Pregabalin and gabapentin have similar advantage and disadvantage. They are well tolerated and have no drug interactions. Dizziness and drowsiness are their primary common adverse effects. The binding affinity of pregabalin for the calcium channels α2-δ1 subunit is 6 times greater than that of gabapentin, which may explains why pregabalin is more clinically effective at lower doses [26]. Due to short half-life, the administration with gabapentin must be frequent. And careful titration is required due to nonlinear absorption of gabapentin [27]. Comparatively, pregabalin is well tolerated, and has predictable absorption across the gastrointestinal tract and linear pharmacokinetics [28].

### Antidepressants

The beneficial effect of antidepressants has been established in various neuropathic pain states. Tricyclic antidepressants (TCAs) and Selective serotonin norepinephrine reuptake inhibitors (SSNRIs) are the main antidepressants used in neuropathic pain treatment. TCAs are efficacious for several types of neuropathic pain including DPN, nerve injury pain, PHN, and central poststroke pain [29]. Their analgesia effects are att**r**ibuted to inhibiting reuptake of serotonin and noradrenaline from presynaptic terminals. TCAs show both analgesia efficacy and antidepressant effect, the pain-relieving effect is independent of their mood-elevating properties [30]. Therefore, TCAs may be a good choice for neuropathic pain patients with co-existing depression. Besides, TCAs are cheap and convenient to be administered. TCAs have several side-effects (eg., dry mouth, constipation, and orthostatichypotension), which are mostly due to their anti-cholinergic and anti-histaminergic properties [29]. The notable side-effect is that TCAs can also inhibit sodium channels to prolong cardiotoxic QTc interval even with therapeutic doses, especially when patients suffer from ischemic cardiac disease or ventricular conduction abnormalities. Therefore, an ECG is mandatory before the start of the treatment [31].

SSNRIs such as duloxetine and venlafaxine have shown consistent efficacy in DPN [32]. They selectively inhibit reuptake of 5-HT and NE from presynaptic membrane in the central nervous system. The advantages of SSNRIs are the same as TCAs. SSNRIs are better tolerated than TCAs since they do not show anticholinergic, antihistaminic, and antiadrenergic side effects [33]. The most common adverse effect is their impact on gastrointestinal tract (eg., nausea and vomiting) [24].

### Topical lidocaine

Topical lidocaine has been demonstrated the efficacious analgesic effect in patients with PHN and allodynia [24]. Despite its analgesia mechanisms is still unknown, it is assumed to block sodium channels so that it can reduce ectopic nociceptive pain signal transmission [34]. Without a relevant systemic absorption, topical application offers a good benefit to risk ratio with mild local reactions (eg., erythema or rash). Therefore, topical lidocaine is particularly suitable for patients with localized peripheral neuropathic pain [24]. Currently, topical lidocaine has not shown any efficacy in central neuropathic pain.

### Opioid analgesics and tramadol

Several RCTs have demonstrated opioids to be effective in relieving pain in PHN, DPN, spinal cord injury and so on [24]. The analgesia effects of opioids are due to inhibiting noxious transmission via μ, κ, δ receptors distributed in the nervous system [35]. However, opioids are not recommended as first-line treatments for patients with neuropathic pain because the side-effects (misuse, constipation, somnolence and drug addiction and diversion) are difficult to be avoided. Clinicians should address risk factors for abuse when patients need to take opioids. In addition, opioids sometimes can induce hyperalgesia, as pronocioceptive systems is also activated after opioid exposure. Guidelines for chronic noncancer pain indicate to use the lowest effective dose of opioids, and to monitor the signs of misuse [36].

Tramadol, a synthetic opioid analogue, is a weak analgesic through binding to the μ opioid receptor. And it also performs analgesic effect by inhibiting serotonin and norepinephrine reuptake. It could provide relatively rapid pain relief in several neuropathic pain conditions including PHN, DPN, and post-amputation pain [24]. Its long-term side effects are similar to strong opioid analgesics. Because of its action on serotonin, tramadol can cause a serotonin syndrome when administrated with serotoninergic drugs [24,37].

## Nonpharmacological management for neuropathic pain

Although pharmacotherapy remains the mainstay of neuropathic pain management, application of medications alone always cannot achieve a sufficient level of pain relief for the patients with neuropathic pain. Therefore, when comprehensive analgesics fail to relieve pain adequately even with maximum doses or side effects of these medications undermine their analgesic effect, other methods (psychological, physical, and surgical treatment) are effective options to support and improve pharmacological therapy. The major nonpharmacological treatments for neuropathic pain are displayed in Table 4.

**Table 4:** Major nonpharmacological treatment of neuropathic pain

Classification	Name
**Psychocorporal treatment**	Cognitive and behavioral techniques
	Operant behavioral therapy
	Self-hypnosis training
**Physical treatment**	Massages
	Joint mobilization
	Transcutaneous electrical nerve stimulation
	Repetitive transcranial magnetic stimulation
	Acupuncture
**Neurosurgical techniques**	
Ablation	Nerve avulsion or section
	Dorsal rhizotomy
	Spinal dorsal root entry zone lesions
	Spinothalamic tractotomies
	Thalamotomies
	Cingulotomy
	Frontal lobotomy
	Destruction of the primary sensory cortex
Neuromodulation	Periperal nerve stimulation
	Spinal cord stimulation
	Deep brain stimulation
	Motor cortex stimulation

### Psychological treatment

Psychological treatment is also important, as neuropathic pain commonly co-occurs with depression, anxiety and poor quality of life [3]. Psychological interventions attempt to change patients' thoughts, strengthen their beliefs and improve the aggressive behavioral responses to pain. As a result, these comorbid conditions induced by neuropathic pain are removed and persistent pain syndrome is relieved indirectly. Cognitive and behavioral techniques, operant behavioral therapy and self-hypnosis training are the primary forms. The effectiveness of psychological managements for neuropathic pain conditions has been proved in a small preliminary study [38]. Basic mechanisms for neuropathic pain triggered emotional and cognitive disorders are unknown.

### Physical treatment

Besides massages, electrophysiotherapy is the primary technology of physical therapies that are also commonly used for alternative pain-relieving treatment. One of the simplest forms of electrophysiotherapy is TENS. A few studies have demonstrated the efficacy of TENS [39]. Therefore, this technology is often used as an ancillary support to the drug or other physical treatments. Another is repetitive transcranial magnetic stimulation (rTMS) which has showed transient efficacy in central and peripheral neuropathic pains [40]. Acupuncture is a kind of traditional medicine commonly used to relieve pain and show to be effective in some cases of neuropathic pain treatments [41]. However, much of basic mechanisms for the analgesic effects induced by acupuncture remain to be investigated.

### Surgical treatment

Neurosurgical interventions contain 2 categories such as nerve ablation and neuromodulation. Ablative interventions are always considered as last choice of treatment because of the damage to the nervous system and modest improvement. Recent studies using animal models indicate that nerve injury may trigger long-term potentiation in sensory synapses in the pain-related cortex [19,42]. Comparatively, neuromodulation (peripheral nerve stimulation, spinal cord stimulation, deep brain stimulation and motor cortex stimulation) do not damage nerve deliberately and is accepted by patients gradually [39]. Spinal cord stimulation (SCS) is one of the common treatments for refractory neuropathic pain [39]. The therapy of SCS is based on the gate control theory of pain, and its effects on pain perception are also related to activation of inhibitory GABA-ergic and cholinergic spinal interneurons [43]. Good evidence for the pain relief effect of SCS has been found in failed back surgery syndrome, complex regional pain syndrome, DPN and peripheral nerve injury [43]. The common side effects of SCS are hardware malfunctionm, wound infection, electrode migration and so on. Likewise, deep brain stimulation and motor cortex stimulation are also invasive procedures that are showed to alleviate chronic neuropathic pain [39]. One possible mechanism for lost-effect of SCS in neurpathic pain conditions is that many of endogenous inhibitory systems may be already activated in the condition of chronic neuropathic pain, thus further stimulation will not produce analgesic effects due to the occlusion.

## Other therapeutic approaches and novel drug targets

### Botulinum toxin

Botulinum toxin (BTX), a neurotoxic protein is synthesized by the bacterium Clostridium botulinum, and there are seven different serotypes designated as A-G. Animal experiments have confirmed that BTX-A can inhibit the secretion of substance P and calcitonin gene-related peptide in cultures of neurons, these results indicate that BTX-A directly suppresses nociceptors which may explain its relief of neuropathic pain symptoms [44]. To assess the benefits of subcutaneous injection of BTX-A for neuropathic pain treatment in clinical practice, several RCT studies have been performed to test the therapeutic benefits. A clinical trial investigated the analgesia effect of BTX-A in 29 patients with focal painful neuropathies and mechanical allodynia, and indicated for the first time to confirm the analgesic efficiency of BTX-A [45]. Recently, a RCT also confirmed the effect of subcutaneous administration with BTX-A, it significantly decreased pain, reduced opioid use compared with lidocaine and placebo [46]. Although there are some positive results for neuropathic pain treatment, more RCTs with larger sample are needed to verify the analgesic effect of BTX-A.

### High-concentration capsaicin Patch

Capsaicin is a highly selective activating ligand for transient receptor potential vanilloid 1 receptor (TRPV1). Interacting with sensory afferents via TRPV1, capsaicin can cause local damage and defunctionalize nociceptive sensory axons to transmit pain information [47]. Despite low-concentration capsaicin is currently recommended as third-line treatment of neuropathic pain, it is troublesome to be applied several times daily with limited efficiency [24]. To avoid the discomfort application and improve the potential therapeutic effect, high-concentration capsaicin patch (capsaicin, 8%) was developed to be used in neuropathic pain treatment. 8% Capsaicin was shown to provide rapid and sustain pain relief between the second and the tenth week after the capsaicin application in a RCT with 402 PHN patients [48]. In addition, application of high-concentration patch produced significantly analgesia effect in patients with painful HIV-associated distal sensory polyneuropathy [49]. Researches on long-term benefits of this treatment found that repeated treatments with high-concentration patch over 48 weeks are generally efficacious, safe and well tolerated in PHN patients, with mild-to-moderate application site erythema, pain, edema, and papules [50]. Epidermal nerve fiber density has shown nearly full recovery 24 weeks after a high dose capsaicin exposure in healthy volunteers [51]. However, this treatment is unlikely to reduce any central potentiation triggered by nerve injury.

### Lacosamide

Lacosamide, synthesized as an anticonvulsive drug, also show antinociceptive effects. It can control neuronal hyperexcitability and modulate collapsin-response mediator protein 2 (CRMP-2) that inhibits a key modulator of pain transmission N-methyl-D-aspartate receptor subunit NR2B [43]. Several clinical trials have confirmed its analgesia effect for painful DPN treatment [52]. Furthermore, long-term safety profile and sustained efficacy of lacosamide have been demonstrated in an open-label follow-on trial followed the DPN patients application of lacosamide up to about 2.5 years [53].

### Combination therapies

Clinically, two or more drugs are often used empirically to achieve more satisfactory pain relief and fewer side effects. However, there are only few studies of combination therapies to provide enough supportive evidence in neuropathic pain treatment. Combination therapy with gabapentin and extended-release morphine in patients with PHN or painful DPN showed improved pain relief with lower doses in comparison with either compound was given alone [54]. The consistent results were also verified by several other Clinical trials such as combination of gabapentin and extended release oxycodone [55], pregabalin and oxycodone [56], pregabalin and topical 5% lidocaine [57], as well as sodium valproate and glyceryl trinitrate spray [58]. Apart from combination of two different drugs, combination therapies with pharmacological and nonpharmacological methods also achieved better analgesic efficacy. Results of a new study about pregabalin and transcutaneous electrical nerve stimulation (TENS) in combination showed a beneficial effect than either alone for painful DPN treatment [59].

## Conclusions and future direction of translational research

Neuropathic pain, often caused by nerve injury, is commonly observed among patients with different diseases. Development and maintenance of chronic neuropathic pain are likely mediated by a series of complex molecular mechanisms. Previous attempts at identifying basic pain mechanisms have focused mainly on neurons in the DRG and spinal dorsal horn, and few synaptic-level studies or new drugs are designed to target the injury-related cortical plasticity that maintains chronic neuropathic pain. As a result, few interventions that solely target neuropathic pain are limited. Recent efforts have begun to strongly tackle cortical synaptic mechanisms mediating neuropathic pain (for example, see Wang et al., 2011 for AC1 as a potential novel target for neuropathic pain), and promising therapeutic interventions targeting cortical molecular mechanisms have begun to emerge. Future research will likely explore specific intracellular inhibitors of plasticity related targets, in contrast to the plethora of nociception related medications currently available.

As a kind of complex diseases, it's difficult to obtain satisfactory analgesic results for neuropathic pain with numerous pharmacological and non-pharmacological therapies. Therefore, clinicians need to consider the advantage and disadvantage of these managements to avoid ineffective treatments, maximize curing proven beneficial in clinical trials, and minimize the side effect of therapies. To improve the current management of patients with neuropathic pain, evidence-based basic studies should be made for pharmacological or non-pharmacological approaches to guide the managements in the future.

## Abbreviations

CPN: Common peroneal nerve; ACC: Anterior cingulate cortex; LTP: Long term potentiation; PKMζ: Protein kinase M-ζ; ZIP: ()ζ-pseudosubstrate inhibitory peptide; TCAs: Tricyclic antidepressants; SSNRIs: Selective serotonin norepinephrine reuptake inhibitors; DPN: Peripheral neuropathy nerve injury pain; PHN: Postherpetic neuralgia; GABA: γ-aminobutyric acid; BTX: Botulinum toxin; TRPV1: Transient receptor potential vanilloid 1 receptor; CRMP-2: Collapsin-response mediator protein 2; TENS: Transcutaneous electrical nerve stimulation; rTMS: Repetitive transcranial magnetic stimulation; SCS: Spinal cord stimulation.

## Competing interests

The authors declare that they have no competing interests.

## Authors' contributions

BX and GD participated in the drafted manuscript. HRY prepared the tables. MZ and YWW organized and revised the manuscript. All authors have read and approved the final manuscript.
